# Microbiological Testing for Coronavirus Disease 2019

**DOI:** 10.31662/jmaj.2021-0012

**Published:** 2021-04-02

**Authors:** Kei Yamamoto, Norio Ohmagari

**Affiliations:** 1 Disease Control and Prevention Center, National Center for Global Health and Medicine, Tokyo, Japan

**Keywords:** COVID-19, microbiological test, nucleic acid detection test, antigen test, Japan

## Abstract

Microbiological diagnosis of coronavirus disease 2019 (COVID-19) is mainly performed through nucleic acid amplification test (NAAT) and antigen test. Although NAAT is the standard diagnostic test, its use is limited by insufficient laboratory resources and long turnaround time. Point-of-care NAAT tests have been introduced to address these shortcomings, but their varied sensitivity and resource constraints remain a concern. Antigen tests require fewer resources but have low sensitivity. Nevertheless, low-sensitivity tests may be useful depending on the situation. In contrast, in some clinical phases of COVID-19, high-sensitivity tests may provide false-negative results. Therefore, the right testing strategy is needed for an accurate diagnosis. In this review, the characteristics and clinical applications of microbiological tests available in Japan (NAAT, antigen test, and antibody test) are discussed. The clinical diagnosis of COVID-19 is slightly complicated, and cases in which the infection spreads from asymptomatic infected individuals are many; hence, laboratory diagnosis is essential to prevent further transmission.

## Introduction

The coronavirus disease 2019 (COVID-19), which became a global pandemic in early 2020, is still raging in Japan and other parts of the world. Although nucleic acid amplification tests (NAAT) are mainly used for definitive diagnosis, various reagents and testing equipment have been introduced and approved for research and *in vitro* diagnostics in Japan ^(1), (2)^. In this review, we describe the specimens for COVID-19 diagnosis, their collection methods, and the characteristics of diagnostic equipment and reagents available in Japan, as well as their use in clinical practice.

NAAT and antigen tests are the microbiological techniques used for the definitive diagnosis of COVID-19 and are covered by insurance in Japan. For NAAT, reverse transcription-polymerase chain reaction (RT-PCR), real-time RT-PCR, loop-mediated isothermal amplification (LAMP), and other isothermal amplification methods have been used. In contrast, as of January 22, 2020, immunochromatography, chemiluminescent enzyme immunoassay (CLEIA), and other assay methods are approved for antigen testing in Japan ^(2)^. Specimens are mainly collected from the respiratory tract, but saliva can also be used. The detection rate of severe acute respiratory syndrome coronavirus 2 (SARS-CoV-2), the causative agent of COVID-19, is higher when using lower respiratory tract specimens, such as sputum and bronchoalveolar lavage fluid, than upper respiratory tract specimens, such as nasopharyngeal swabs; however, the upper respiratory tract specimens and saliva are often used because they are easier to collect ^(3)^. Additionally, antibody tests can be outsourced in Japan; however, these tests are not covered by insurance. Notably, the detection of antibodies varies according to the COVID-19 phase, limiting the reliability of antibody tests for acute diagnosis of COVID-19. Next-generation sequencing could also be used to diagnose COVID-19; however, it is only suitable for research purposes, not clinical practice. Moreover, virus culture is a diagnostic technique for infectious disease and is limited to biosafety level 3 research facilities in Japan and other countries; thus, we have not discussed the details of virus culture in this paper.

## Specimen Collection And Handling and Infection Control during Collection

Nasopharyngeal swabs are collected by swabbing followed by suspension in virus transport media (VTM), inactivating agents, or reagents specifically designed for the test. If the storage period is short (less than 24 h), suspension in saline or storage as a swab may be acceptable. However, considering that RNA quickly degrades and may result in false-negative results, favorable storage conditions should be maintained. Nasal vestibular and nasopharyngeal swabs have comparable sensitivity and should be handled in a similar way. Saliva and sputum should be placed in a collection container with or without an inactivating agent and stored until processing. Stool and urine can also be used for viral diagnosis, although their clinical significance is unknown.

When collecting nasopharyngeal swabs and sputum, sufficient safety measures are necessary to prevent contact infection and exposure to droplets and aerosols. Therefore, medical personnel should wear surgical or N95 masks, including face guards and partitions ^(4)^. However, nasal vestibular swabs have a low risk of inducing sneezing or coughing and are not painful or invasive; hence, the patients themselves can collect these. Their self-sampling sensitivity is comparable to that of samples collected by a medical professional ^(3)^. The nasal vestibular swabs should be collected by the patient under observation by a medical professional, and medical staff should use a surgical mask and gloves only ^(4)^. For saliva collection, the collection container may be contaminated; hence, medical staff should disinfect it. Since the viral load decreases after gargling ^(5)^, the Japanese guidelines recommend saliva collection at least 10 minutes, preferably more than 30 minutes, after eating, drinking, brushing, and gargling ^(4)^.

## NAAT

NAAT detects viruses by amplifying the targeted nucleic acids and is used to confirm the presence of viruses that are difficult to culture, making it the gold standard test for COVID-19 microbiological diagnosis. However, if the amount of nucleic acid is insufficient, the test can be negative, regardless of its sensitivity. The nasopharynx is the standard specimen for NAAT, but the nasal vestibule or middle turbinate swab can be used if self-collection is possible. Saliva is also widely used for testing, although it requires additional processing before NAAT. The use of lower respiratory tract specimens is recommended for patients with a high likelihood of the disease based on clinical manifestations, such as persistent symptoms with typical pulmonary lesions ^(3)^. In Japan, SmartGene, FilmArray, GeneXpert, and ID NOW are available as point-of-care testing (POCT) ^(1), (2)^; the former three use RT-PCR, and ID NOW is based on isothermal amplification.

### RT-PCR

**Figure 1.**
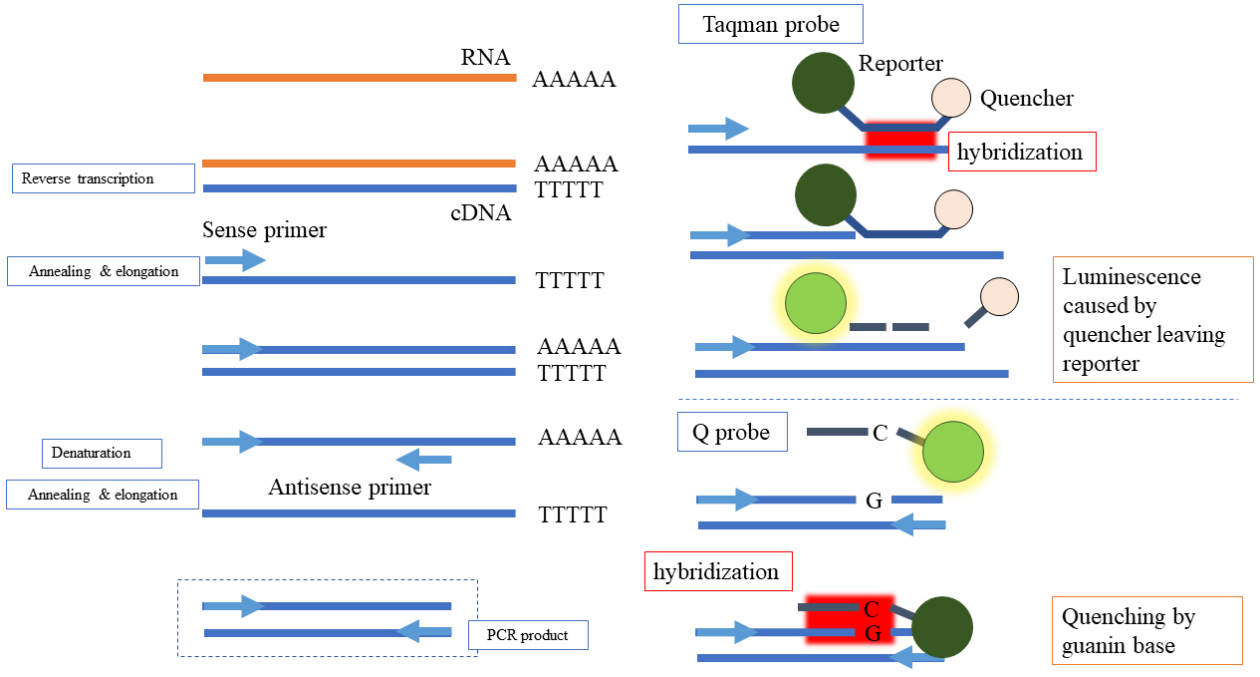
The measurement principle of real-time reverse transcription polymerase chain reaction.

RT-PCR is a molecular technique that detects and quantifies the nucleic acid of RNA viruses such as SARS-CoV-2. In this case, the RNA is first converted to complementary DNA, and with the use of primers, a specific region of the DNA is amplified through a temperature-regulated process. The nucleic acid region defined by the primer is labeled by a probe, and the amplification can be captured by a fluorescent signal in real time (Figure 1). The point at which the nucleic acid amplification curve exceeds a certain threshold is called the threshold cycle (Ct) value. The Ct value is proportional to the amount of targeted nucleic acid in the sample; therefore, the copy number can be calculated by creating a calibration curve using a sample with a known copy number of the targeted virus or nucleic acid. The testing process includes specimen pretreatment, nucleic acid extraction, and nucleic acid amplification reaction. However, nucleic acid extraction and amplification are time-consuming processes and are difficult to perform in many hospital laboratories. Various RNA extraction techniques, such as direct RT-PCR or fully automated testing equipment, are available in Japan (Table 1). The sensitivity and specificity of RT-PCR for SARS-CoV-2 in various specimens, based on nasopharyngeal RT-PCR as the gold standard, are indicated in Table 2 ^(3)^; oropharyngeal swabs have low sensitivity and are unsuitable for use. In the early phase of the pandemic, the sensitivity of PCR was reported to be approximately 70% ^(6)^, and this low sensitivity was due to the use of oropharyngeal swabs. In addition, the peak viral shedding of COVID-19 occurs from the day before the onset of illness to about 2-3 days after the onset of illness ^(7)^, and the results of RT-qPCR tend to vary in the later stages of illness ^(8), (9)^. Therefore, the timing of the test is essential in the interpretation of sensitivity reports. The sensitivity of the test in the early stages of the disease is often high, which is approximately 90% according to a meta-analysis ^(10)^. Notably, the sensitivity of repeat RT-PCR is higher than that of a single test (88% vs. 71%) ^(3)^; however, the accuracy of the sensitivity tests may be limited by variances in timing and specimen type and the reduced repeat tests ^(11)^. The total reaction time of the POCT instrument ranges between 50 and 70 min. RT-PCR POCT with SmartGene ^(1)^, FilmArray ^(12)^, and GeneXpert ^(13), (14)^ have sensitivity equivalent to that of the conventional RT-PCR method. GeneXpert displays the Ct value, and the SmartGene displays the alternative number of cycles; however, FilmArray does not show the Ct value and cannot estimate the viral load. Nevertheless, FilmArray can concurrently assay other respiratory pathogens.

**Table 1.** Nucleic Acid Amplification Tests Available in Japan (January 22, 2021).

Methods	Product name	Sales company in Japan	Turnaround time	Approved as *in vitro* diagnostics	Measuring device
Quantitative reverse transcription-polymerase chain reaction (RT-qPCR)	2019 novel coronavirus (nCoV) fluorescence real-time RT-PCR kit	Sysmex Corporation	90 min, except time to extract nucleic acid	Yes	
	MEBRIGHT SARS-CoV-2 Kit	MEDICAL ＆ BIOLOGICAL LABORATORIES CO., LTD.	90 min, except time to extract nucleic acid	Yes	
	SARS-CoV-2 GeneSoC N2 Kyorin	Kyorin Pharmaceutical Company, Limited	15 min, except time to extract nucleic acid	No	GeneSoC
	SARS-CoV-2 RT-qPCR Detection Kit Ver. 2	FUJIFILM Wako Pure Chemical Corporation	50 min, except time to extract nucleic acid^*^1	No	
Direct RT-qPCR	Ampdirect 2019-nCoV Detection Kit	SHIMADZU CORPORATION	70 min	Yes	
	SGNP nCoV/Flu PCR Detection kit	SUDx-Biotec Corporation	20-60 min	Yes	
	Takara SARS-CoV-2 direct PCR detection kit	Takara Bio Inc.	60 min	Yes	
	TaqPath SARS-CoV-2 real-time PCR detection kit HT	Life Technologies Japan Ltd.	60 min	Yes	
	SARS-CoV-2 Detection Kit -Multi-	TOYOBO CO., LTD.	60 min	No	
	SARS-CoV-2 gene detection kit KYOKUTO Ver. 2	KYOKUTO PHARMACEUTICAL INDUSTRIAL CO., LTD.	60 min	No	
	SARS-CoV-2 detection kit	KUBIX Inc.	60 min	No	
	KANEKA Direct RT-qPCR Kit "SARS-CoV-2"	KANEKA CORPORATION	60 min	No	
	GENECUBE HQ SARS―CoV-2	TOYOBO CO., LTD	35 min	Yes	GENECUBE
	Cobas SARS-CoV-2 ＆ Flu A/B	Roche Diagnostics K.K.	180 min (96 tests)	Yes	Cobas 6800
					Cobas 8800
	i-densy Pack SARS-CoV-2	ARKRAY Factory, Inc.	80 min	Yes	i-densy IS-5320
	BD Max SARS-CoV-2	Nippon Becton Dickinson Company, Ltd.	120-180 min (24 tests)	No	BD Max
	ELITe MGB SARS-CoV-2 Kit	Precision System Science Co., Ltd.	150 min (12 tests)	No	ELITeInGenius
	VIASURE SARS-CoV-2 Kit	Precision System Science Co., Ltd.	90-120 min (8 tests)	No	geneLEADVIII
	μTAS Wako COVID-19	FUJIFILM Wako Pure Chemical Corporation	75 min	No	μTAS Wako g1
Point-of-care test (POCT) RT-qPCR	Xpert Xpress SARS-CoV-2 “Cepheid”	Beckman Coulter, Inc.	45 min	Yes	GeneXpert
	FilmArray Respiratory 2.1 panel	bioMérieux Japan Ltd.	45 min	Yes	FilmArray
	SmartGene SARS-CoV-2 detection kit	MIZUHO MEDY Co., Ltd.	70 min	No	SmartGene
Isothermal amplification	Loopamp SARS-CoV-2 detection kit	EIKEN CHEMICAL CO., LTD.	35 min, except time to extract nucleic acid^*^2	Yes	Loopamp EXIA^*^3
	SmartAmp SARS-CoV-2 detection kit	K.K.DNAFORM.	30 min, except time to extract nucleic acid	Yes	
Direct isothermal amplification	SARS-CoV-2 RNA detection kit TRCReady SARS CoV 2 i	TOSOH CORPORATION	40 min	Yes	TRCReady-80
	SARS-CoV-2 detection kit LAMPdirect Genelyzer KIT	CANON MEDICAL SYSTEMS CORPORATION	30 min	No	Genelyzer FIII Genelyzer FII Genelyzer F-MS
	Aptima SARS-CoV-2	Hologic Japan, Inc.	210 min (275 tests/8 hours)	Yes	Panther system
POCT isothermal amplification	ID NOW SARS-CoV-2	Abbott Diagnostics Medical Co., Ltd.	13 min	Yes	ID NOW

“Direct” is defined as the process of nucleic acid extraction that is included in the reagents or test equipment, and “POCT” is defined as the process that does not require dispensing of reagents using micropipettes, etc., and can be handled by physicians at the bedside. Many product names are not written in English, so they have been translated by the author and are not the official product names. Turnaround time is based on the time indicated by each manufacturer and is only a rough guide because some specimen preparation times are not included. ^*^1 Nucleic acid extraction time can be reduced using “SARS-CoV-2 Lysis Buffer Ver. 2, FUJIFILM Wako Pure Chemical Corporation” (time required, 15 min). ^*^2 The rapid extraction reagent “Virus RNA extraction kit, EIKEN CHEMICAL CO.” is available. ^*^3 Visual judgment is also possible. RT-qPCR, quantitative reverse transcription polymerase chain reaction; SARS-CoV-2, severe acute respiratory syndrome coronavirus 2; POCT, point-of-care test

**Table 2.** Sensitivity and Specificity of Nucleic Acid Amplification Tests in Each Specimen Based on Nasopharyngeal Swab as a Reference.

	Sensitivity	Specificity
Saliva collected from patients without cough	0.90 (95% confidence interval (CI), 0.85-0.93)	0.98 (95% CI, 0.93-1.00)
Saliva collected from patients with cough	0.99 (95% CI, 0.94-1.00)	0.96 (95% CI, 0.83-0.99)
Oropharyngeal swab	0.76 (95% CI, 0.58-0.88)	0.98 (95% CI, 0.96-0.99)
Nasal vestibular swab	0.89 (95% CI, 0.83-0.94)	1.00 (95% CI, 0.99-1.00)
Middle turbinate swab	0.95 (95% CI, 0.83-0.99)	1.00 (95% CI, 0.89-1.00)
Nasal vestibular and oropharyngeal swab	0.95 (95% CI, 0.69-0.99)	0.99 (95% CI, 0.92-1.00)

Reproduced from Hanson KE, Caliendo AM, Arias CA, et al. The Infectious Diseases Society of America Guidelines on the Diagnosis of COVID-19: molecular diagnostic testing. Clin Infect Dis. 2021:ciab048 ^(3)^ with permission.

### LAMP and other isothermal amplification methods

Unlike RT-PCR, the isothermal amplification method does not require temperature changes. Nucleic acid amplification is relatively quick, and many test kits can provide results in less than an hour, excluding nucleic acid extraction (Table 1). For instance, the nicking enzyme amplification reaction (NEAR) method does not involve complex elongation reactions and shows exponential nucleic acid amplification, making the amplification reach a plateau within a few minutes ^(15)^. Despite the need for dedicated measurement equipment for most techniques, visual color changes are sufficient for positive judgment in the LAMP method. Although the LAMP and SmartAmp methods require a nucleic acid extraction process, a simplified extraction reagent is available for the Loopamp SARS-CoV-2 detection kit. It has a detection sensitivity of more than 100 copies/test, although its detectability has only been validated using pseudoviruses ^(16)^. A non-peer-reviewed report indicates that LAMP using this simple extraction kit has a high false-positive rate ^(17)^. The simple heat-denaturing extraction method is less effective than the conventional nucleic acid extraction using GeneAnalyzer ^(18)^. Although nucleic acid extraction is required for the transcription-mediated amplification method, a fully automated system (Panther system) allows for high-throughput testing ^(19)^. Detection kits using transcription reverse transcription concerted reactions and NEAR methods available in Japan can be directly performed from the specimen. In particular, ID NOW, which uses the NEAR method, can provide test results within 13 min; hence, it is faster than the antigen test described below. However, the IDSA guidelines recommend that patients with a high pretest probability should be confirmed with other NAAT tests although they have a negative result with ID NOW ^(3)^, because of reports of the lower sensitivity of ID NOW (approximately 70%) than RT-PCR ^(20), (21), (22), (23)^. Since specimens that test negative for ID NOW and positive for RT-PCR have a low viral load ^(21), (22)^, these tests may be useful in screening for infectiousness.

## Antigen Test

The antigen test measures some of the viral proteins and, similar to NAAT, indicates the presence of pathogens. Several antigen tests have been approved in Japan for insurance coverage. Despite the lower sensitivity of the antigen test compared with the PCR method ^(20)^, it is simple to perform and has a shorter turnaround time than most NAATs. Therefore, it is a suitable test for low-resource centers. In Japan, antigen tests are based on immunochromatographic methods, in which antigens are captured by antibodies solidified on filter paper, and the CLEIA method, in which antibodies are bound to magnetic particles, magnetized, and then reacted with a luminescent reagent to measure the amount of luminescence. As of January 22, 2021, Japan had also approved other two methods of SARS-CoV-2 rapid antigen test: Rapiim SARS-CoV-2-N and LumiraDx SARS-CoV-2 Ag. The former is based on a protein analysis method that detects light scattering when antigens bind to antibodies and microparticles solidified on a light-waveguide membrane, while the latter is a microfluidic immunofluorescence method characterized by antigen-antibody reaction with antigen-capturing particles placed in a microfluidic channel and fluorescent labeling.

The CLEIA is quantitative, and the amount of antigens correlates with the viral load ^(24), (25)^. This technique sets a cutoff value that determines the positive or negative result. In addition to upper respiratory tract specimens, such as nasopharyngeal and nasal vestibular secretions, saliva specimens can be used for the CLEIA antigen test ^(26), (27)^. The immunochromatographic method is simple, does not require sophisticated measuring equipment, and has a short reaction time of 15-30 min. The SARS-CoV-2 rapid antigen tests available in Japan are Espline SARS-CoV-2, QuickNavi SARS-CoV-2, ImmunoAce SARS-CoV-2, and Panbio COVID-19 Antigen Rapid test. Rapid antigen tests have a higher virus detection limit than NAAT. In Japan, it is recommended that rapid antigen tests be used on days 2-9, the period when the viral load is likely to be high ^(4)^. Nasopharyngeal or nasal swabs are commonly used as specimens, but saliva specimens are not warranted due to poor diagnostic performance ^(28), (29)^.

The use of nonhuman antibodies from rodents for the antibody-antigen reactions is limited by possible nonspecific binding between labeled and solidified triggered by highly viscous and heterophilic antibodies ^(30)^, resulting in false positives. False-positive cases of Espline SARS-CoV-2 are a problem in Japan ^(31), (32)^. Although the cause of the false-positive results is unclear, viscosity may be involved because the number of specimens negative for RT-PCR and positive for antigen was significantly lower in the specimens developed in VTM than in those developed in the processing reagent (0/229 vs. 2/40, p = 0.02, Fisher’s exact test) ^(33)^. In addition, since diagnosis is made by human eyes, judging when the test line is subtle may be difficult. As illustrated by rapid tests for influenza, the diagnostic performance of these tests may be low ^(34)^. Although the performance of each rapid antigen test kit is different, the Espline has a relatively high positive concordance rate (72%) in the early course of illness (2-9 days) and a low positive concordance rate (22%) after 10 days of illness ^(33), (35)^. Among samples with at least 10^2^ copies/test (8.5 copies/μL), the positive concordance rate of Espline was 92%, and the kappa coefficient was 0.86, indicating high concordance with RT-PCR 2-9 days after the onset of symptom ^(33)^. Rapiim SARS-CoV-2-N has a high concordance rate with RT-PCR for relatively low-copy specimens because the test is performed using dedicated equipment. However, similar to Espline ^(33)^, Rapiim SARS-CoV-2-N had a low positive concordance rate for specimens with less than 100 copies/test ^(36)^. LumiraDx SARS-CoV-2 Ag, however, has a high concordance rate with RT-PCR (kappa coefficient, 0.9), even in specimens with low viral load at late disease date ^(37), (38)^. In contrast, LumiraDx SARS-CoV-2 Ag results in many positive cases that are negative in RT-PCR (nasopharynx, 11%; nasal cavity, 7%) ^(37)^. Therefore, the positive results of LumiraDx should be cautiously interpreted, especially when the patient has a low prior probability of COVID-19, such as the absence of a typical pulmonary lesion or contact with a COVID-19 patient. However, positive and negative judgments in specimens with low viral load differ depending on the RT-PCR method ^(39)^, and the rate of false-positive results in this kit is inconclusive.

## Antibody Test

**Figure 2.**
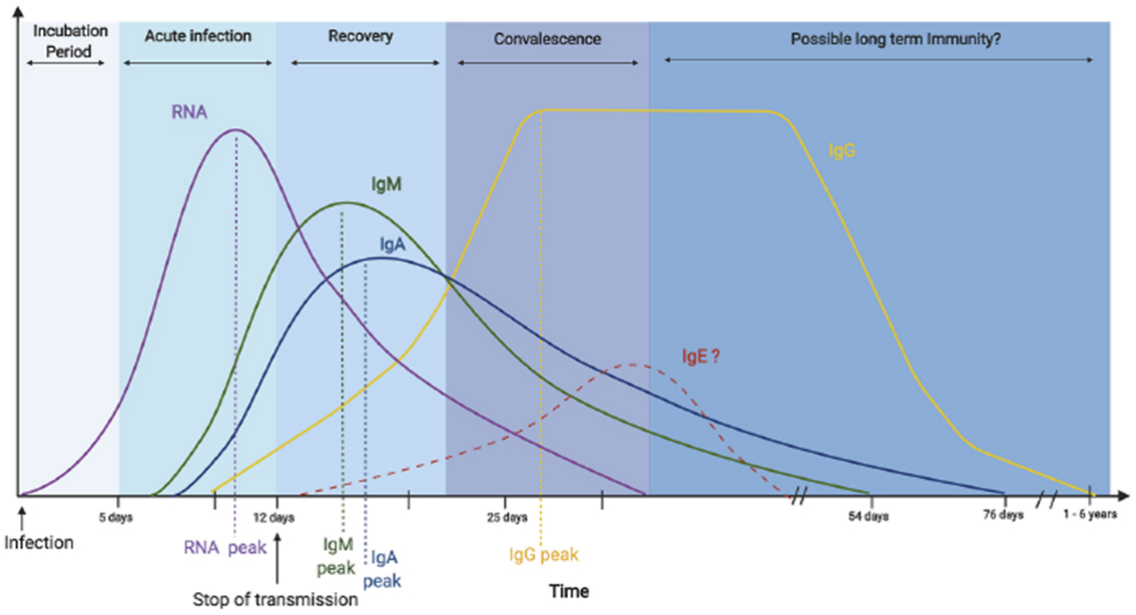
The serological course of coronavirus disease 2019. Reproduced from Galipeau Y, Greig M, Liu G, et al. Humoral responses and serological assays in SARS-CoV-2 infections. Front Immunol. 2020;11:610688 ^(40)^ with permission. ^©^ 2020 Galipeau, Greig, Liu, Driedger and Langlois.

The use of antibodies for the definitive diagnosis of COVID-19 is limited, and antibody tests are not covered by insurance in Japan. Notably, IgM levels begin to rise approximately 7 days after the onset of illness and peak at around 14 days after the onset of illness (Figure 2) ^(40)^. There are many antibody-testing methods, but immunochromatographic antibody testing should not be used because of possible cross-reactivity with other antibodies and low correlation with neutralizing antibodies ^(41)^. Enzymatic chemiluminescence immunization and chemiluminescence immunization, which have been shown to correlate with neutralizing antibodies, should be used ^(40)^. Tests that depend on enzyme-based chemiluminescence immunization or chemiluminescence immunization are available for research. The sensitivity and specificity of these tests are quite low at 0-7 days of illness, 33.3% and 33.3%, respectively, but high after 15 days of illness, 96.7% and 93.3%, respectively ^(42)^.

## How to Use Testing for SARS-CoV-2 in Clinical Setting

Based on this review, the tests that should be used for the acute diagnosis of COVID-19 are NAAT or antigen tests. The significance of the acute diagnosis can be divided into two main categories: 1) assessment of infectiousness and 2) detection of pathogen and implementation of therapeutic intervention.

Regarding the assessment of infectiousness, during the early stages of COVID-19, the viral load is high, hence raising infectiveness ^(7), (43)^. An accurate diagnosis is necessary; however, diagnosing COVID-19 based on clinical manifestations alone is difficult because symptoms are quickly alleviated in young and immunocompetent individuals. NAAT screening tests, such as POCT, can be uniformly performed in facilities with abundant testing resources, but many facilities have not embraced NAAT because of high resource demands and long turnaround time. Besides, delays in reagent supply and the high cost of maintaining the laboratory equipment hinder the use of NAAT. Despite the overemphasis of the low sensitivity of the rapid antigen tests, they have high detection sensitivity in the early stages of the disease, when the viral load tends to be high. Therefore, after the 10th day of illness, when the rapid antigen test is not suitable, a person with COVID-19 may not be infectious due to reduced viral load^(43), (44)^. Therefore, screening by antigen testing may be sufficient in preventing the spread of infection^(45)^. In a preprint paper, the rates of positive viral culture in the early stages of the disease in antigen-positive and antigen-negative cases were 96% and 20%, respectively ^(46)^. In a report using the rapid antigen test approved in Japan in combination with viral culture, mainly in early-stage specimens, the positive results of viral culture in antigen-positive and antigen-negative cases were 88% vs. 30% for the Espline, 100% vs. 38% for QuickNavi, and 100% vs. 27% for ImmunoAce ^(47)^. Since some culture-positive cases were found in antigen-negative cases, this may not be a perfect screening method; however, it may help screen asymptomatic patients, although it is not approved by the Japanese government ^(4)^.

**Figure 3.**
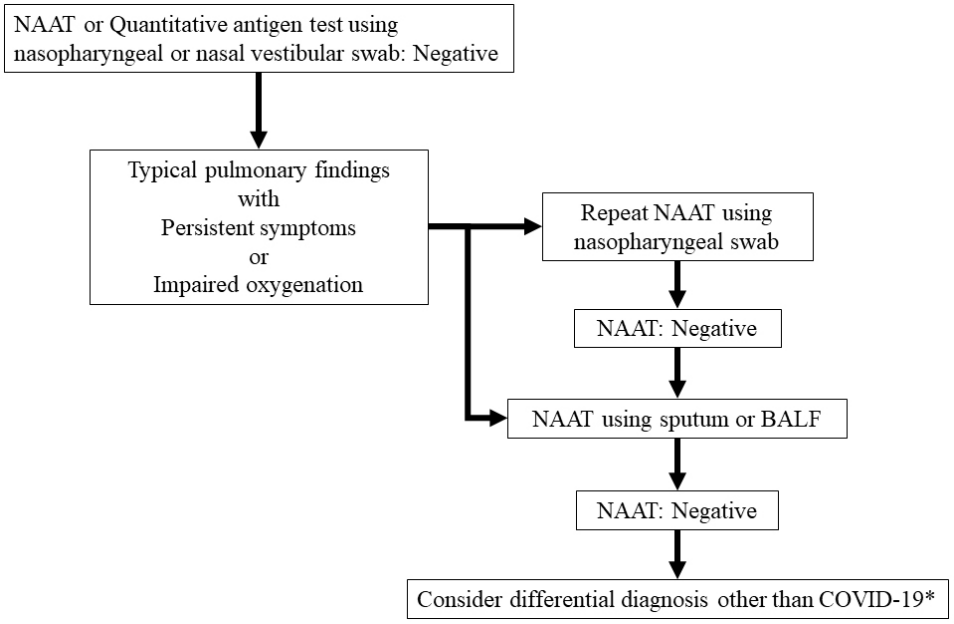
Repeat testing strategy for the patients of coronavirus disease 2019 who need aiding in therapeutic intervention. NAAT, nucleic acid amplification tests; BALF, bronchoalveolar lavage fluid. *Consider antibody testing, if necessary.

In contrast, NAAT and quantitative antigen testing are necessary for detecting the pathogen and aiding in therapeutic intervention. COVID-19 causes severe disease 7-10 days after onset ^(48)^, and the amount of virus shed begins to decline^(8), (9)^. In such cases, repeated NAAT using lower respiratory tract specimens should be considered, as shown in Figure 3 ^(3)^. Antibody testing may be considered in some cases, although it is not often necessary.

In Japan, COVID-19 cases diagnosed by antibody testing are not mandatorily reported to the government; moreover, the antibody levels start increasing between days 7 and 14 after the onset of symptom ^(40)^, when the individual is no longer infectious. Although the antibody test has limited clinical applications, it may be useful for epidemiological studies, determining the cause of symptoms when the virus is undetectable by NAAT and antigen tests such as MIS-C ^(49)^, and evaluating long-term complications ^(50)^.

## Conclusions

The clinical diagnosis of COVID-19 is slightly complicated, although the acute diagnosis is significant to assess for infectiousness to prevent the spread of the disease and detect the virus to facilitate therapeutic intervention. With regard to infection prevention, in many cases, the infection spreads from asymptomatic infected individuals; therefore, laboratory diagnosis is essential for preventing further virus transmission. NAAT and antigen tests are the mainstay of diagnosis, although NAAT is highly sensitive and has resource limitations. Antigen testing is less sensitive than NAAT, but it has relatively high sensitivity and proves to be less resource-intensive for diagnosis during periods of high infectivity. Depending on the laboratory resources, using antigen testing as a screening test for SARS-CoV-2 infection may be reasonable. However, if there are treatment indications and a definitive diagnosis is to be made, the more sensitive NAAT should be selected; furthermore, the use of lower respiratory tract specimens and repeat testing should sometimes be considered. Newly approved tests have both advantages and pitfalls; this necessitates better characterization of the tests to inform the clinical application of tests based on appropriate risk assessment in the context of the likelihood of infections.

## Article Information

### Conflicts of Interest

K.Y. received research grants from Fujirebio, Inc., Mizuho Medy, Co., Ltd., and VisGene Inc.; N.O. received grants from Sanofi Pasteur and Eiken Chemical Co., Ltd., outside the submitted work.
